# *In Vitro* Antioxidant and Antidiabetic Potentials of *Syzygium caryophyllatum* L. Alston

**DOI:** 10.1155/2020/9529042

**Published:** 2020-07-28

**Authors:** Herath Pathiranage Thathmi Wathsara, Hasitha Dhananjaya Weeratunge, Mohamed Naeem Ahammadu Mubarak, Pahan Indika Godakumbura, Pathmasiri Ranasinghe

**Affiliations:** ^1^Department of Chemistry, Faculty of Applied Sciences, University of Sri Jayewardenepura, Nugegoda, Sri Lanka; ^2^Herbal Technology Section (HTS), Modern Research & Development Complex (MRDC), Industrial Technology Institute (ITI), 503A Halbarawa Gardens, Malabe, Sri Lanka; ^3^Residue Analysis Laboratory, Industrial Technology Institute (ITI), 363, Bauddhaloka Mawatha, Colombo 07, Sri Lanka

## Abstract

*Syzygium caryophyllatum* L. Alston (Family: Myrtaceae, Sinhala: Heendan) is a red-listed plant that has been used in traditional medicine in Sri Lanka for the treatment of diabetes, but it is yet to be exploited for its potential uses as a functional food or a source of supplements. The present study focused on the evaluation of antidiabetic property of *S. caryophyllatum* fruits and leaves assessing antioxidant, antiglycation, and antiamylase activities and functional mineral element composition. The crude extracts (CR) of leaves and fruits were fractionated into hexane (Hex) ethyl acetate (EA) and aqueous (AQ) and evaluated for bioactivities along with the crude extracts. The isolated fraction (C_3_) of Hex fraction of fruit showed significantly high (*p* < 0.05) antiamylase activity with IC_50_ value 2.27 ± 1.81 *μ*g/mL where the Hex fraction of fruits exhibited the IC_50_ value as 47.20 ± 0.3 *μ*g/mL which was higher than acarbose (IC_50_: 87.96 ± 1.43 *μ*g/mL). The EA fraction of leaves showed highest values for DPPH radical scavenging activity, ferric reducing antioxidant power, and oxygen radical absorbance capacity. Significantly high (*p* < 0.05) ABTS radical scavenging activity and iron chelating activity were observed in Hex fraction of fruit. The composition of volatiles in leaf oil was studied with GC-MS, and 58 compounds were identified. Inductively coupled plasma-mass spectrometry data revealed the presence of biologically significant trace elements such as Fe, Zn, Mg, Cu, Se, and Sr in leaves and fruits. It is concluded that the Hex fraction of *S. caryophyllatum* fruits will be a good source for the formulation of supplements for diabetic management with further evaluation of potency and efficacy.

## 1. Introduction

The prevalence of diabetes mellitus has shown a rapid increase over the years, and according to the reports of the WHO, individuals suffering from diabetes mellitus worldwide will show a marked increase of up to 592 million in 2035 from 422 million in 2014. Further in 2015, 1.6 million deaths were estimated to be directly or indirectly caused by diabetes. At present, developing countries are much affected by the more predominant form of diabetes—Type 2 diabetes that accounts for increased morbidity and disability causing a threat to the economic growth in most developing countries. However, reports revealed that more than 80% of deaths resulting from diabetes in developing countries are due to rapid urbanization and poor healthcare facilities [1].

Diabetes Mellitus (DM) is a metabolic disorder of multiple etiology characterized by chronic hyperglycemia which is mainly caused by one or combination of factors including the ineffectiveness of insulin, deficiency of insulin secretion, and insulin resistance of the cells, in particular, skeletal muscle tissues [2]. Changes in food patterns and lifestyle such as high intake of calories, less exercise, and physiological stress conditions are also closely associated with the development and progression of DM [3].

Prolonged hyperglycemic conditions in diabetes patients induce nonenzymatic glycation, which is a reaction between the amino group of proteins and the carbonyl group of reducing sugar to form a fluorescent, Advanced Glycation End-products (AGEs) [4]. Apart from elevated sugar levels in the blood, exposure to exogenous free radicals and consumption of processed foods with a high content of fructose may also accelerate the accumulation of AGEs [5]. Studies have proven that hyperglycemia and signal-transducing receptor interactions of AGEs formation induce increased production of free radicals leading to oxidative stress [6, 7]. This in turn leads to various pathological conditions, such as cancer, neurological disorders, atherosclerosis, hypertension, type 2 diabetes, acute respiratory distress syndrome, idiopathic pulmonary fibrosis, chronic obstructive pulmonary disease, and asthma [6].

Studies have further revealed that the imbalance of several essential trace elements leads to the development and progression of DM. Further clinical studies have suggested that patients with diabetes have an increased risk of trace element deficiency. Hence, it is recommended to increase the dietary intake of some trace elements or as a dietary supplement to manage this condition during DM [8]. The mineral elements play a pivotal role in numerous basic physiological functions of the human body and the deficiency thus leads to a variety of extensive diseases and disorders [9]. Several mineral elements have been identified as the cofactors of antioxidative enzymes and play an important role in protecting the insulin-secreting pancreatic *β*-cells [8]. Thus management of type 2 DM includes reduction of postprandial glucose concentrations by inhibition of *α*-amylase and *α*-glucosidase enzymes which delays the digestion of carbohydrates, thus, lowering the hyperglycemic condition.

In recent years, the usage and the vitality of natural products have created great curiosity among the scientific community. This is mainly due to the benefits of natural products such as high effectiveness, low risk of side effects, low cost, and abundant availability.

Ethnobotanical information reveals that there is a wide array of plants that possessed potential antidiabetic properties and antioxidant properties [10, 11]. In this regard, over 200 pure compounds from herbal plants are scientifically proven to have hypoglycemic activity [12].

*Syzygium* is a genus from the Myrtaceae family possessing 1200–1800 species. *Syzygium caryophyllatum* (*Sinhala*: Heendan) is one of the species that has been classified as endangered species under the International Union for Conservation of Nature (IUCN) and is native to Sri Lanka and India [13]. In India, *S. caryophyllatum* plant has been studied for its antibacterial and antioxidant activities using some *in vitro* antioxidant assays [14]. However, this is the 1^st^ Sri Lankan study to report the antioxidant activities and antidiabetic activities of *S. caryophyllatum*. In traditional folklore medical practices, the decoction of *S. caryophyllatum* is used in the treatment for ailments of diabetes mellitus [15]. In Sri Lanka, *S. caryophyllatum* is considered as an underutilized wild fruit amidst all its beneficial properties due to the lack of reported data on its nutritional value, poor awareness on the beneficial roles of the fruit, upcoming modern agricultural practices, and over imported fruits [16].

Therefore, the present study aimed to evaluate *in vitro* bioactivities such as antioxidant, antiamylase, and antiglycation and quantify mineral elements and volatile oil compositions of *Syzygium caryophyllatum* in Sri Lanka. Furthermore, the outcome of this study will be used to determine whether *Syzygium caryophyllatum* fruits and leaves are potential sources of dietary supplement in managing DM and its complications.

## 2. Materials and Methods

### 2.1. Chemical and Reagents

Folin-Ciocalteu reagent (FC Reagent), gallic acid, quercetin, acarbose, 6-hydroxy-2-5-7-8-tetramethylchroman-2carboxylic acid (Trolox), ethylenediaminetetraacetic acid (EDTA), 1,1-diphenyl-2-picryl-hydrazyl (DPPH), 2, 2-azino-bis (3ethylbenzothiazoline-6-sulfonic acid) diammonium salt (ABTS), potassium persulphate, 2, 2′-azobis (2-amidinopropane) dihydrochloride (AAPH), sodium fluorescein, 2,4,6 tripyridyl-s-triazine (TPTZ), and *α*-amylase (*Bacillus amyloliquefaciens*) were purchased from Roche Diagnostics (USA), and 3,5-dinitrosalycylic acid, 4, 4′-disulfonic acid sodium salt (ferrozine), soluble starch, bovine serum albumin (BSA), D-glucose, and trichloro-acetic acid (TCA) were purchased from Sigma-Aldrich (USA). All other chemicals used for the preparation of buffers and solvents were in the analytical grade. Spectrophotometric assays were conducted using 96-well microplate readers (Spectra Max Plus384, Molecular Devices, USA, and SPECTRAmax-Gemini EM, Molecular Devices Inc., USA). The mineral analysis was done using microwave digester CEM Mar 5, USA, using the Easy Prep digestion program and ICP-MS (Agilent 7900, USA).

### 2.2. Plant Materials

**Figure 1:**
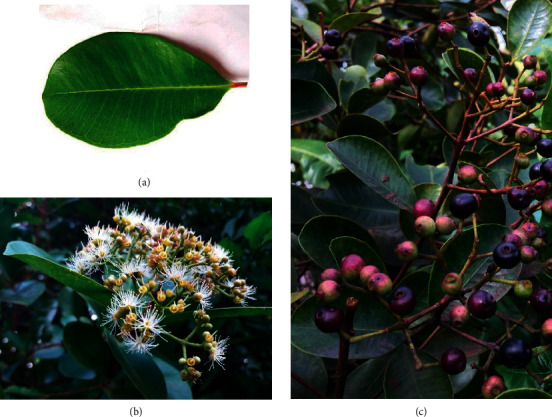
Different parts of the *Syzygium caryophyllatum* plant: (a) leaf, (b) flowers, and (c) fruits.

Fresh leaves and ripened fruits (Figure 1) were collected into sterile polypropylene bags from the areas of Homagama and Malabe (Colombo district, Sri Lanka) during the period from April to May 2016. The plant materials were botanically identified by the botanist, Dr. Chandima Wijesiriwardena, Principle Research Scientist, Industrial Technology Institute, Sri Lanka, and voucher specimens (leaves WF10-1 and fruits-WF10-2) were deposited at the Herbal Technology Section (HTS), Industrial Technology Institute (ITI), Sri Lanka. The collected materials were washed with running water followed by distilled water, cleaned, air dried, and ground to obtain a fine powder. The powder was packed in polypropylene bags and stored at −20°C for further analyses.

### 2.3. Preparation of Extracts

#### 2.3.1. Preparation of Leaf Crude Extract of *Syzygium caryophyllatum*

The moisture content of the powdered leaves was recorded by moisture analyzer (moisture 13.53%). The powdered sample (225 g dry weight of sample) was extracted by the reflux apparatus for 3 hours and filtered using a muslin cloth and later with Whatman number 1 filter paper. The filtrate was treated with 70% ethanol (1 : 3, extract: ethanol) and centrifuged to remove the precipitate. The extract was concentrated in a vacuum below 50°C and neutralized to pH 7 with 0.01 M HCl.

#### 2.3.2. Preparation of Fruit Crude Extract of *Syzygium caryophyllatum*

The ripen fruits (30 g dry weight of sample) without seed were extracted with ethanol by overnight cold extraction with stirring. The ethanol extract was concentrated in a vacuum at 40°C.

The crude extracts of leaves and fruits were fractionated into hexane and ethyl acetate by using separation funnels, and all concentrated samples were stored at −20°C prior to the analyses. The extracts were evaporated to dryness by rotary evaporator, and a known amount of sample was dissolved in DMSO for further analyses.

### 2.4. Compound Isolation from Hexane Fraction of *Syzygium caryophyllatum* Fruits

The fraction was separated using column chromatography with different organic solvents as the mobile phase. A column of 75 cm height and 2.5 cm diameter was packed with silica gel 100–200 mesh with hexane as a solvent in the wet packing method. Compound isolation was carried out in two different methods by using different solvents on the isolation procedure.

#### 2.4.1. Hexane and Ethyl Acetate Solvents on Isolation Procedure (Hex and EA)

The 50 mg of Hex fraction of fruits was separated by column by increasing polarity of eluent hexane and ethyl acetate 100 : 0 and 0 : 100, respectively. The two separated isolates were tested for *α*-amylase inhibition activity.

#### 2.4.2. Hexane, Dichloromethane, and Ethyl Acetate Solvents on Isolation Procedure (Hex, DCM, and EA)

Column chromatography was performed using 200 mg of Hex fraction of fruits to obtain fractions by increasing polarity using a gradient elution technique as follows: Fractions (FR) 1–10 (Hex), FR 11–12 (Hex : DCM = 3 : 1), FR 13 (Hex : DCM = 2 : 1), FR 14–19 (DCM), and FR 20–22 (DCM : EA = 95 : 5) to obtain 51.8 mg (C_1_: combined fractions 1–13), 95.7 mg (C_2_: combined fractions 14–19), and 6.8 mg (C_3_: combined fractions 20–22), respectively. All combined fractions were tested for *α*-amylase inhibition activity.

**Figure 2:**
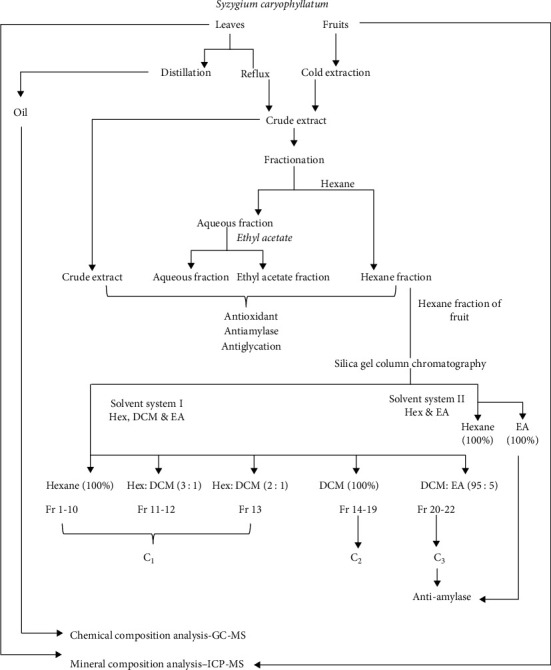
Flow chart depicting the methodology of the study.

Flowchart of the overall methodology is shown in Figure 2.

Leaves and fruits of *Syzygium caryophyllatum* plant were extracted, fractionated, and subjected for phytochemical analysis, antioxidant, antiamylase, antiglycation potentials analysis, and compounds isolation. Chemical compositions of *Syzygium caryophyllatum* leaf oil were analyzed by GC-MS and mineral composition of leaves and fruits were analyzed by ICP-MS.

### 2.5. Estimation of Phytochemical Compounds

#### 2.5.1. Determination of Total Polyphenol Content (TPC)

The total polyphenol content (TPC) was determined using the Folin-Ciocalteu (FC) reagent method [17] with minor modification using 96-well microplates. Freshly prepared 110 *μ*L of FC Reagent was added to 20 *μ*L of the sample. The mixture was treated with 10% sodium carbonate and incubated at room temperature for 30 min and absorbance was measured at 765 nm. Gallic acid was used as a reference standard, and the results were expressed as milligram gallic acid equivalent per gram of sample (mg GAE/g of sample).

#### 2.5.2. Determination of Total Flavonoid Content (TFC)

The total flavonoid content (TFC) was determined using an aluminum chloride colorimetric method using 96-well microplates [18]. Extracts were dissolved in methanol, and 100 *μ*L of the sample was mixed with 100 *μ*L of 2% aluminum chloride. The mixture was incubated at room temperature for 10 min and absorbance was measured at 415 nm. Quercetin was used as a reference standard, and the results were expressed as milligram quercetin equivalent per gram of sample (mg QE/g of sample).

### 2.6. Evaluation of Antioxidant Activity

#### 2.6.1. Ferric Reducing Antioxidant Power (FRAP) Assay

The assay method was based on the reduction of Fe^3+^ to Fe^2+^ by the electron donation ability of antioxidants resulting in an intense blue-colored complex (Fe^2+^-tripyridyltriazine) [18]. The FRAP reagent was prepared freshly by mixing acetate buffer (300 mM, pH 3.6) solution of TPTZ in HCl and FeCl_3_ (20 mM) in 10 : 1 : 1 ratio and incubated at 37°C. Two hundred microliters of the reaction mixture (FRAP reagent and sample) was incubated at room temperature, and absorbance was measured at 600 nm. Vitamin E analogue Trolox was used as the reference standard where the results were expressed as mg of Trolox equivalent per gram of extract (mg TE/g of extract).

#### 2.6.2. DPPH Radical Scavenging Assay

The radical scavenging activity was determined by using 1, 1-diphenyl-2-picryl- hydrazyl (DPPH) stable radical method [18]. The method is based on the reduction of purple-colored 1, 1-diphenyl-2-picrylhydrazyl to a yellow-colored diphenylpicrylhydrazine. The reaction volume of 200 *μ*L containing sample (50 *μ*L), methanol, and DPPH were incubated at room temperature for 10 min, and absorbance was measured at 517 nm. A percentage inhibition versus concentration was plotted, and the concentration of the sample required for 50% inhibition was determined and represented as EC_50_ value. The following formula was used to calculate the percent inhibition:

(1) $$\begin{array}{c} \%\text{Radical scavenging activity}=\frac{\left(C_{\text{AB}}-S_{\text{AB}}\right)}{C_{\text{AB}}}\times 100, \end{array}$$

where *C*_AB_ is the absorbance of the control, and *S*_AB_ is the absorbance of the sample.

#### 2.6.3. ABTS Radical Scavenging Activity

The ABTS^+^ radical cation (ABTS^+^) scavenging activity assay was performed [19] with some modifications in 96-well microplates. Plant extracts were prepared in phosphate buffer solutions and were allowed to react with ABTS^+^ radicals in a microwell plate for 10 min. Absorbance was measured in 734 nm and Trolox was used as the standard. The following formula was used to calculate the percent inhibition:

(2) $$\begin{array}{c} \%\text{Radical scavenging activity}=\frac{\left(C_{\text{AB}}-S_{\text{AB}}\right)}{C_{\text{AB}}}\times 100, \end{array}$$

where *C*_AB_ is the absorbance of the control, and *S*_AB_ is the absorbance of the sample.

The antiradical activity was expressed as EC_50_, the concentration required to cause 50% inhibition of ABTS^+^ radicals.

#### 2.6.4. Oxygen Radical Absorbance Capacity (ORAC) Assay

The ORAC assay is a hydrogen atom transfer- (HAT-) based assay which measures the depletion of fluorescence intensity of a fluorescent probe upon the generation of peroxy radicals due to the oxidation of azo compounds. In the presence of an antioxidant compound, the decaying of fluorescent intensity is retarded. The overall decay is calculated by the difference between the area under the fluorescence decay curve (AUC) of the sample of interest and AUC of blank [20]. The reaction volume of 200 *μ*L (pH 7.4) containing fluorescein (100 *μ*L) and sample (50 *μ*L) was preincubated at 37°C for 5 min. The 2,2′-azobis (2-amidinopropane) dihydrochloride (AAPH) solution was added (50 *μ*L) to the mixture, and the plate (black 96 well plates) was immediately placed in a fluorescent microplate reader (SPECTRAmax-Gemini EM, Molecular Devices Inc, USA). The decay of fluorescence was recorded every minute for 35 min, and the excitation and emission wavelengths were obtained at 494 nm and 535 nm, respectively. Phosphate buffer (75 mM, pH 7.4) replacing the sample was used as the blank, and Trolox was used as the standard antioxidant. Results were expressed as mg of Trolox equivalent per gram of extract.

#### 2.6.5. Ferrous Ion Chelating Activity

The ferrous ion chelating (FIC) activity was measured by the decrease in the absorbance at 562 nm of the iron (II)-ferrozine complex [21]. EDTA was used as the reference standard, and all the samples and standards were prepared in distilled water. The sample was mixed with 1 mM ferrous sulfate and distilled water followed by 1 mM ferrozine solution. The ability of the sample to chelate ferrous ions was calculated relative to the control (distilled water instead of the sample) by the following formula. The results were expressed as mg EDTA equivalent per gram of extract.

(3) $$\begin{array}{c} \%\text{Chelating activity}=\frac{\left(C_{\text{AB}}-S_{\text{AB}}\right)}{C_{\text{AB}}}\times 100, \end{array}$$

where *S*_AB_ is the absorbance of the sample and *C*_AB_ is the absorbance of the control.

### 2.7. Determination of *α*-Amylase Inhibition Activity

The *α*-amylase inhibition activity was carried out using the dinitrosalicylic (DNS) method [22], where the inhibition activity was measured by quantifying the maltose liberated under the assay conditions. The enzyme inhibitory activity was expressed as a decrease in units of maltose liberated. A modified dinitrosalicylic acid (DNS) method was adopted to estimate the maltose equivalent. The reaction volume containing 40 *μ*L of 1% (w/v) starch solution and 775 *μ*L of 100 mM sodium acetate buffer was preincubated with 135 *μ*L of the sample at 40°C shaking water bath for 10 min. The mixture was further incubated with 50 *μ*L of the *α*-amylase enzyme (5 *μ*g/mL) at 40°C shaking water bath for 15 min. The reaction was terminated by adding 500 *μ*L DNS reagent, and the mixture was placed in a boiling water bath for 5 min followed by cooling in an ice bath. Absorbance was measured at 540 nm using a 96-well microplate reader. Antiamylase activity (inhibition %) was calculated using the following equation:

(4) $$\begin{array}{c} \text{Inhibition}\left(\%\right)=\frac{\left[A_{\text{C}}-\left(A_{\text{S}}-A_{\text{b}}\right)\right]}{A_{\text{C}}}\times 100, \end{array}$$

where *A*_C_ is the absorbance of the control, *A*_S_ is the absorbance of the sample, and *A*_b_ is the absorbance of the sample blank.

### 2.8. BSA-Glucose Glycation Inhibitory Activity Assay

Antiglycation activity assay was performed with some modifications to the previous methodology [23] for CR extracts of leaves and fruits of *Syzygium caryophyllatum*. A mixture of 800 *μ*g/mL BSA, 400 mM glucose, and 80 *μ*L of the sample in 50 mM phosphate buffer (pH 7.4) containing sodium azide (0.02%) was incubated at 60°C for 40 h. The 600 *μ*L of the reaction mixture was transferred to 1.5 mL microcentrifuge tubes and 60 *μ*L of 100% (w/v) TCA was added, and sample mixtures were centrifuged at 15,000 rpm at 4°C for 4 min. The resultant advanced glycation end products-BSA (AGEs-BSA) precipitate was then dissolved in 1 mL of phosphate buffer saline (pH 10), and the fluorescence intensity was measured at an excitation and emission wavelengths of 370 nm and 440 nm using a 96-well fluorescence microplate reader (SpectraMax, Gemini EM, Molecular Devices, Inc., USA). Rutin was used as the positive control and antiglycation activity (% inhibition) was calculated using the following equation followed by the calculation of IC_50_ value:

(5) $$\begin{array}{c} \text{Inhibition}\left(\%\right)=\frac{\left[F_{\text{c}}–F_{\text{b}}\right]–\left[F_{\text{s}}–F_{\text{sb}}\right]}{\left[F_{\text{c}}–F_{\text{b}}\right]}\times 100, \end{array}$$

where *F*_c_ is the fluorescence of the control, *F*_b_ is the fluorescence of BSA, *F*_s_ is the fluorescence of the sample, and *F*_sb_ is the fluorescence of the sample blank.

All the above *in vitro* bioassay procedures were validated at Herbal Technology Section, Industrial Technology Institute, Sri Lanka (ISO: 9005 certified Laboratory).

### 2.9. Gas Chromatography-Mass Spectrometry (GC-MS) Analysis

The extracted essential oil from the leaves was subjected to GC-MS analysis to identify volatile compounds. The analysis was carried out by using TRACE 1300 coupled to ISQ QD mass spectrophotometer instrument (Thermo Fisher Scientific, Milan, Italy). The sample was injected into Thermo-scientific TG-WAX capillary column (30 m × 0.25 mm × 0.25 *μ*m) fused with silica having polyethylene glycol as the stationary phase with helium as the carrier gas (1 mL/min). The temperature of the injector was maintained at 250°C. The GC oven temperature was initially programmed at a temperature of 60°C and was ramped up to 220°C at the rate of 5°C/min. GC-MS interface temperature was maintained at 250°C. The mass detector was operated in EI mode in a scan mass range of *m*/*z* 40–450. The identification of compounds was ascertained by comparing the mass spectral values with the known compounds in NIST 11, USA mass spectral database.

### 2.10. Preparation of Plant Extracts for Mineral Content Determination

Approximately 0.5 g of homogenized sample was weighed into easy prep high-pressure microwave vessel, and 10.0 mL of 65% nitric acid was added. The easy prep microwave digestion program was followed to digest the samples by using a microwave digester (CEM MARS 5, USA). The digested sample was quantitatively transferred and filtered using No. 542 Whatman filter paper followed by washing with deionized water and volume up to 25 mL. The prepared solution was used to analyze mineral content by ICP-MS (Agilent 7900, USA).

### 2.11. Statistical Analysis

Statistical analysis was performed using Graph Pad Prism (version 7). All experimental data was analyzed by one-way analysis of variance (ANOVA). ANOVA's Turkey multiple comparison test was used to compare the mean values. Pearson‘s correlation coefficient was used for the correlation analysis, and in all cases, *p* values less than 0.05 (*p* < 0.05) were considered statistically significant. The quantitative results of phytochemicals, antioxidant potentials, and enzyme inhibition values were expressed as mean ± standard deviation (SD).

## 3. Results

*S*. *caryophyllatum*, fruits, and leaves were extracted according to the method described above and fractionated into nonpolar and polar solvents with increasing polarity, hexane, and ethyl acetate, respectively. This solvent–solvent partitioning procedure was allowed to generate hexane (Hex) fraction, ethyl acetate (EA) fraction, and aqueous (AQ) fraction which was labelled and stored in −20°C prior to analysis. The yield was estimated to the dry weight, which ranged from 0.6 mg to 31.2 g, where the highest yield was recorded in crude (CR) extract of fruits (31.2 g) and the lowest was recorded in Hex fraction of leaves (0.6 mg). The weights of the CR extracts, Hex, EA, and AQ fractions are given in Table 1, and the yield was calculated as a percentage.

All the crude extracts and fractions were subjected to further analysis except the hexane fraction of leaves of *Syzygium caryophyllatum* due to the unavailability of adequate sample yield.

### 3.1. Total Polyphenol Content (TPC) and Total Flavonoid Content (TFC) of Leaves and Fruit Extracts of *Syzygium caryophyllatum* Plant Extracts

Total polyphenol content was expressed as milligram gallic acid equivalent per gram of sample (mg GAE/g) and the total flavonoid content as milligram quercetin equivalent per gram of sample (mg QE/g). Total polyphenol values varied from 0.0923 mg GAE/g of sample to 8.18 mg GAE/g of sample and from 1.72 mg GAE/g of sample to 8.92 mg GAE/g of sample for leaves and fruits, respectively. Polyphenol contents of samples were differed significantly (*p* < 0.05).  Table 2 presents the TPC and TFC values for crude and fractions of samples. The highest TPC value was recorded in CR extract of fruits followed by CR extract of leaves and AQ fraction of leaves. Flavonoid content of the tested samples varied significantly (*p* < 0.05) among extracts. TFC ranged from 0.001 mg QE/g of sample to 2.03 mg QE/g of sample, and the highest TFC value was reported in CR extract of fruits with value as 2.02 ± 0.19 mg QE/g of the sample followed by Hex fraction and EA fraction of fruits with values 0.80 ± 0.05 mg QE/g of sample and 0.37 ± 0.01 mg QE/g of sample, respectively.

### 3.2. *In Vitro* Antioxidant Properties of Different Extracts of *Syzygium caryophyllatum*

The antioxidant properties of crudes and fractions of leaves and fruits of *Syzygium caryophyllatum* were determined using DPPH, ABTS, ORAC, FRAP, and iron chelation assay methods. The results obtained are depicted in Table 3 where the significant differences (*p* < 0.05) were observed between CR extracts of leaves and fruits and among different fractions of leaves and fruits.

#### 3.2.1. DPPH Radical Scavenging Activity

**Figure 3:**
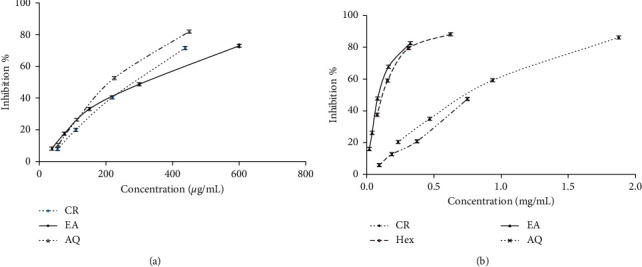
Dose-response curves for DPPH radical scavenging activity of *Syzygium caryophyllatum*: (a) leaves and (b) fruits (CR = crude, Hex = hexane, EA = ethyl acetate, AQ = aqueous).

Results revealed that the crude extract and fractions of *S. caryophyllatum* have the ability to scavenge radicals and depicted a dose-response relationship. DPPH radical scavenging activity of different fractions of *S. caryophyllatum* differed significantly (*p* < 0.05). The EA fraction of leaves exhibited the lowest EC_50_ value followed by Hex and EA fraction of fruits with values 91.2 ± 4.28 *μ*g/mL, 100.97 ± 8.19 *μ*g/mL, and 135.53 ± 9.83 *μ*g/mL, respectively. The results obtained from the fractions showed that EA fractions of leaves and Hex fractions of fruits exhibited high antioxidant activities compared to other fractions. It shows that the accumulation of a variety of antioxidant compounds depends on the polarity of the solvent. Therefore, the compounds responsible for the high radical scavenging activity of *S. caryophyllatum* leaves are mainly medium polarity compounds. The dose-response relationships of *S. caryophyllatum* leaves and fruits are given in Figures 3(a) and 3(b).

#### 3.2.2. ABTS^+^ Radical Scavenging Activity

**Figure 4:**
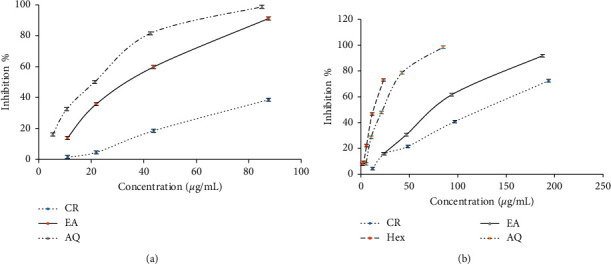
Dose-response curves for ABTS + radical scavenging activity of *Syzygium caryophyllatum*: (a) leaves and (b) fruits (CR = crude, Hex = hexane, EA = ethyl acetate, AQ = aqueous).

The highest radical scavenging activity was reported in Hex fraction of fruits with EC_50_ value 15.01 ± 0.65 *μ*g/mL followed by AQ fraction of *S. caryophyllatum* leaves and AQ fraction of *S. caryophyllatum* fruits, and the EC_50_ values were 27.03 ± 2.06 and 31.45 ± 1.43 *μ*g/mL, respectively. It was found that fruits and leaves showed a significant difference (*p* < 0.05) among fractions. The CR extracts and fractions of leaves and fruits showed significant difference and dose-dependent inhibition on ABTS radical scavenging activity (Figures 4(a) and 4(b)).

#### 3.2.3. Ferric Reducing Antioxidant Power (FRAP)

The CR extracts of leaves and fruits differed significantly (*p* < 0.05) in their FRAP values: CR extract of leaves: 48.27 ± 8.11 mg TE/g of extract and CR extract of fruits: 6.81 ± 1.17 mg TE/g of extract. The FRAP values of the fractions ranged from 14.81 mg TE/g of the extract to 270.41 mg TE/g of extract. EA fraction of leaves was reported as the highest among the tested fractions in leaves and fruits, where the AQ fraction of fruits exhibited the lowest values. These results suggested that EA fraction of leaves and fruits exhibited the highest ferric reducing property among tested fractions, which explains the important behavior of the polar compounds in its reducing property.

#### 3.2.4. Oxygen Radical Absorbance Capacity (ORAC)

The ORAC values of fractions ranged from 17.23 mg TE/g of the extract to 347.32 mg TE/g of extract. The highest activity was recorded in EA fraction of leaves following the AQ fraction of leaves and the lowest was recorded in AQ fraction in fruits. The CR extract of leaves exhibited a significantly higher (*p* < 0.05) activity compared to the CR extract of fruits with values 221.00 ± 34.96 and 45.98 ± 2.58 mg TE/g of extract, respectively.

#### 3.2.5. Iron Chelating Activity

The highest iron chelating ability of CR extract was reported in leaves with values 33.2 ± 1.68 mg EDTA/g of extract and fruits were reported as 13.73 ± 0.69 mg EDTA/g of extract. The Hex fraction of fruits exhibited the highest iron-chelating ability among the tested fractions with value 74.82 ± 2.66 mg EDTA/g of extract, and the lowest was reported in EA fraction of fruits with value 8.28 ± 0.40 mg EDTA/g of extract, respectively. The AQ fraction of *Syzygium caryophyllatum* fruits did not show a significant chelating activity where the highest concentration (2.15 mg/mL) exhibited 16.05% inhibition.

### 3.3. Correlation Analysis between Polyphenol Content and DPPH Radical Scavenging Activity of *Syzygium caryophyllatum* Fruits

**Figure 5:**
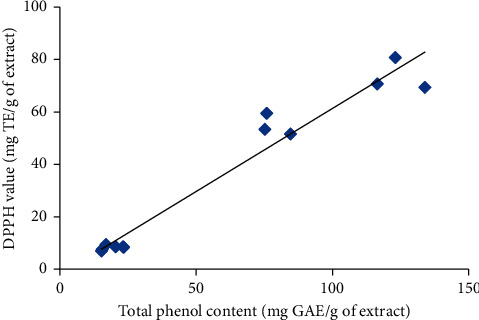
Correlation of *Syzygium caryophyllatum* fruits TPC versus DPPH values (*p* < 0.05).

The correlation coefficient between TPC content and DPPH of *Syzygium caryophyllatum* fruits exhibited a significant (*p* < 0.05) positive correlation with Pearson *r* value 0.9921 (Figure 5).

### 3.4. The *α*-Amylase Inhibitory Activity

**Figure 6:**
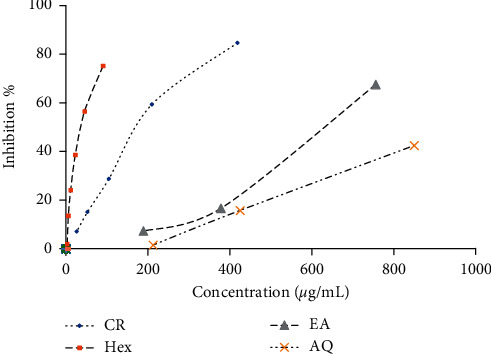
Dose-response relationship of crude and fractions of *Syzygium caryophyllatum* fruits for alpha-amylase inhibitory activity (CR = crude, Hex = hexane, EA = ethyl acetate, AQ = aqueous).

The *α*-amylase inhibition potentials of CR extract, Hex, EA, and AQ fraction of leaves and fruits were analyzed. Fruits exhibited significant inhibition of *α*-amylase activities where the leaves did not exhibit significant inhibitory activities. The Hex fraction of *S. caryophyllatum* fruits was reported to have the highest inhibitory activity with IC_50_ value 47.20 ± 0.3 *μ*g/mL followed by CR extract, EA fraction, and AQ fraction with IC_50_ values 218.26 ± 5.38 *μ*g/mL, 618.85 ± 18.55 *μ*g/mL, and 1014.55 ± 85.55 *μ*g/mL, respectively. Interestingly, the Hex fraction of fruits demonstrated potent *α*-amylase inhibition activity (IC_50_; 47.20 ± 0.3 *μ*g/mL) compared to the standard drug acarbose (IC_50_; 87.96 ± 1.43 *μ*g/mL). The percentage of *α*-amylase inhibitions of crudes and fractions was plotted against concentration (Figure 6) where it showed a dose-dependent increase in percentage activity of CR extract and three types of fractions of fruits.

#### 3.4.1. The *α*-Amylase Inhibitory Activity of Isolated Fractions of Hexane Fraction of *Syzygium caryophyllatum* Fruits

*(1) Hexane and Ethyl Acetate Solvents on Isolation Procedure*. The inhibition potential of compound mixtures of EA fraction was evaluated for *α*-amylase inhibitory activity. The highest tested concentration was 200 *μ*g/mL followed by 100 *μ*g/mL, 50 *μ*g/mL, and 25 *μ*g/mL. The similar inhibition potentials were observed for the first three concentrations, and the % inhibition values were 57.63 ± 0.18%, 55.99 ± 1.60%, and 57.30 ± 0.18%, respectively, where the inhibition potential of 25 *μ*g/mL was observed as a rapid drop to the inhibition 12.78 ± 0.45%. Further inhibition potentials were evaluated between 50 *μ*g/mL and 25 *μ*g/mL (Table 4). The *α*-amylase inhibitory activity of the tested fraction showed a poor linear response (*R*^2^ = 0.6384); hence, IC_50_ was not calculated.

*(2) Dichloromethane and Ethyl Acetate Solvents on Isolation Procedure*. The *α*-amylase inhibitory activity of different combined fractions was analyzed (C_1_, C_2_, and C_3_). The inhibitory activities of C_1_ and C_2_ fractions did not exhibit significant inhibitory activities where the fraction C_3_ showed significant (*p* < 0.05) inhibitory activity for the screened concentrations. The fraction C_3_ was dose-dependent and IC_50_ value was 2.27 ± 1.81 *μ*g/mL.

The summary of *α*-amylase inhibitory activity of hexane fraction and its chromatographic fraction is given in Table 5.

### 3.5. Antiglycation Activity

The antiglycation activity of crude extracts of leaves and fruits was screened for three concentrations 200, 100, and 50 *μ*g/mL (Table 6). The CR extract of leaves exhibited the highest inhibitory activity (93.61 ± 8.53% at a concentration of 200 *μ*g/mL) followed by CR extract of *S. caryophyllatum* fruits (47.97 ± 2.63%). The standard drug rutin exhibited antiglycation activity with IC_50_ value as 34.23 ± 3.18 *μ*g/mL. The IC_50_ value of CR extract of leaves was 57.83 ± 7.91 *μ*g/mL. However, for fruits, similar inhibitions were observed for different doses; hence, IC_50_ value could not be calculated.

### 3.6. Chemical Compositions of *Syzygium caryophyllatum* Leaf Essential Oil

**Figure 7:**
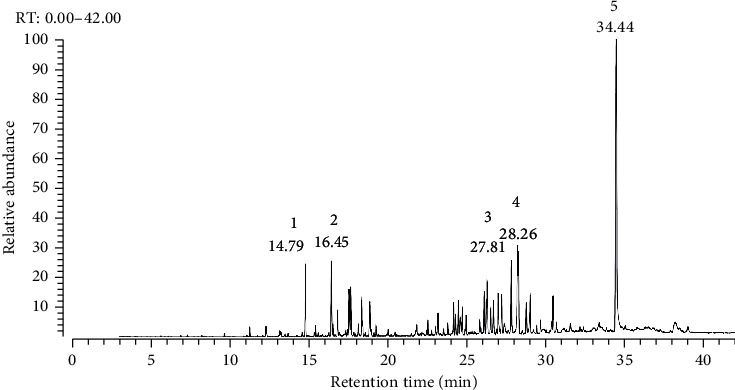
GC-MS chromatogram of *Syzygium caryophyllatum* leaf essential oil.

*Syzygium caryophyllatum* leaves yielded essential oil upon hydrodistillation. GC-MS analysis identified 58 compounds from the essential oil and the retention time and area % of the identified major peaks are listed in Table 7. The major compounds identified in the leaves were phytol with a 24.66% peak area (retention time: 34.44), *α*-cadinol with a 3.87% peak area (retention time: 27.81), *α*-guaiene with a 3.84% peak area (retention time: 26.29), cyclosiolongifolene, 9,10-dehydro with a 3.82% peak area (retention time: 28.26), humulene with a 3.36% peak area (retention time: 16.45), and caryophyllene with a 3.21% peak area (retention time: 14.79), respectively. The GC-MS chromatogram of *Syzygium caryophyllatum* leaf oil is presented in Figure 7.

### 3.7. Mineral Compositions

Functionally important microminerals such as selenium (Se), iron (Fe), strontium (Sr), manganese (Mn), cobalt (Co), copper (Cu), and the toxic heavy metals such as arsenic (As), cadmium (Cd), chromium (Cr), and lead (Pb) were evaluated (Table 8). The ICP-MS data ranged from 0.09 to 4537.0 mg/kg of sample. Sodium (Na) was found to be the highest (leaves: 4357.68 mg/kg of the sample) while Se was found to be the lowest (fruits: 0.09 mg/kg of the sample). The leaves were found to have the highest concentration of the tested minerals compared to the fruits. The second richest macroelement was found to be K (leaves: 3559.25 mg/kg and fruits: 2944.28 mg/kg) followed by Mg (leaves: 2580.40 mg/kg) and Ca (leaves: 1843.52 mg/kg). The maximum amount of Al, Mn, Fe, and Zn was found to be in leaves with values 524.45 mg/kg, 298.82 mg/kg, 221.98 mg/kg, and 21.60 mg/kg, respectively. Among the tested microelements, Sr was found to be the richest with a value of 32.889 mg/kg in leaves which is significantly (*p* < 0.05) different from fruits (2.70 mg/kg). The Cu and Cr were found in similar small concentrations in leaves (Cu: 4.91 mg/kg and Cr: 4.03 mg/kg) and fruits (Cu: 5.23 mg/kg and Cr: 4.65 mg/kg). The maximum concentration of Se was determined to be 0.85 mg/kg in leaves and the fruits exhibited the lowest concentration with a value of 0.09 mg/kg. The investigation of the level of Co revealed that the maximum concentration of the element exists as 0.128 mg/kg in leaves while in the fruits it was not detected at the detection limit of 0.05 mg/kg.

The analysis of toxic heavy metals revealed the presence of a maximum concentration of Pb in leaves (1.02 mg/kg) and the lowest in fruits (0.15 mg/kg). Besides, the concentrations of toxic heavy metals such as Cd and As were not detected in leaves and fruits (limit of detection: 0.05 mg/kg).

## 4. Discussion

Diabetes mellitus (DM) is a complex metabolic disorder characterized by hyperglycemia which is mainly associated with malfunctions and abnormalities in carbohydrate, protein, and fat metabolism. Digestion and absorption of carbohydrates to the blood stream also have a significant influence on DM. Further, the increased production of reactive oxygen species (ROS) under DM accelerates the protein glycation process [24]. The production and accumulation of glycated proteins have shown a strong association with complications of DM [25]. Thus, the management of DM is directed toward addressing multiple targets including reduction of postprandial carbohydrate levels in the blood, improvement in the homeostatic and metabolic process of carbohydrates and lipids, an improvement on the efficiency of antioxidant defense, and control of protein glycation [26].

In this regard, WHO recommends functional ingredients and supplements with multiple functional properties including antidiabetic and the antiradical properties in the control of the DM [27].

In this study, functional properties including *α*-amylase inhibition, protein glycation inhibition, and antioxidant activities of *S. caryophyllatum* fruit and leaf extracts have been investigated along with chemical constituents and mineral analyses. The results revealed the potential of *S. caryophyllatum* as a source of supplement for DM management. To the best of our knowledge, the present study is the first study reported in Sri Lanka on functional activities of *S. caryophyllatum* fruit and leaf extracts. Crude extracts of both fruits and leaves showed significant antioxidant and moderate antiglycation activities. However, only fruit extracts showed *α*-amylase inhibitory activity. Even though both fruits and leaves CR were tested against *α*-glucosidase enzyme inhibitory activity, none of them showed a significant inhibitory activity at a maximum dose of 400 *μ*g/mL (data not shown). Solvent–solvent partitioning of both CR was used to separate and valuate the most potent fractions with *α*-amylase inhibition activity.

Hexane fraction of fruit extract exhibited the highest inhibition activity with IC_50_ value 47.20 ± 0.37 *μ*g/mL and is significantly higher compared to acarbose (IC_50_: 87.96 ± 1.43 *μ*g/mL). Therefore, it was further separated by column chromatography. The fraction C_3_, which was isolated on elution with DCM : EA, 95 : 5, exhibited a significant (*p* < 0.05) *α*-amylase inhibitory activity with an IC_50_ value of 2.27 ± 1.81 *μ*g/mL. The compound or the mixture of compounds responsible for the *α*-amylase inhibition activity may be in the C_3_ compound mixture which is required to be subjected to further isolation and identification. Inhibition may be due to the presence of potential alpha-amylase inhibitors such as alkaloids, tannin, polyphenols, and flavonoids. These inhibitors can also act as starch blockers as they block the hydrolysis of 1,4,-glycosidic linkages of starch and oligosaccharides [28]. Hex fraction of *S. caryophyllatum* fruit extract which can be easily prepared even at a commercial scale may be a very good source for the formulation of supplements for DM management.

Hyperglycemic conditions in DM promote the formation of AGEs that leads to the complications of DM. *In vivo* studies have revealed the involvement of potent glycation inhibitors in the prevention of diabetic complications [29]. In this regard, studies show that pyridoxamine slows down nephropathy in DM, and aminoguanidine reduces the development of albuminuria in diabetic rats [30]. Further, protocatechualdehyde (PCA) has been shown to improve the development of lens opacity (cataract) with a positive effect on glycemic control. PCA was found to be 80% more effective than aminoguanidine in preventing AGE-related complications of DM [30].

However, there are no available reports on the antiglycation activity of *S. caryophyllatum* leaves and fruits. The results of the present study also demonstrate moderate activity for CR extracts for leaf and weak activity for fruits.

Dietary antioxidants are essential to strengthen the oxidative defense system of the body particularly under hyperglycemic conditions. In this regard, the antioxidant activity of *S. caryophyllatum* fruits and leaves was evaluated using four different methods which are having different modes of action.

Free radical scavenging methods such as DPPH and ABTS were used and EC_50_ values of both assays suggested potent radical scavenging activity of *S. caryophyllatum* fruits and leaf extracts. The DPPH, nitrogen centered stable free radical, has been used to determine the free radical scavenging activity. The reduction of DPPH in methanol leads to discoloration from violet to yellow by either hydrogen or electron donation.

A previous study on DPPH radical scavenging activity of the *S. caryophyllatum* leaves has reported DPPH inhibition activity in methanol extract with 81.97%, followed by EA (78.69%) and Hex fraction (34.34%) at the concentration of 100 *μ*g/mL [14]. Annadurai et al. [31] have reported DPPH scavenging activity for the EA fraction of *S. caryophyllatum* leaves as 86.14% inhibition at a concentration of 400 *μ*g/mL. Subramanian et al. [14] and the present study show similar results for the EA fraction with a value of 72.92% inhibition at the concentration of 150 *μ*g/mL. A previous study carried out by sequential extraction method for leaves and fruits has reported having IC_50_ value for DPPH scavenging activity in methanol as 34.9 ± 1.2 *μ*g/mL and 69.4 ± 0.7 *μ*g/mL, respectively, which showed high activity compared to the present study [15].

The scavenging activity against cationic radical indicates the capability of crude and fractions to act as electron or hydrogen donors. Previous studies have reported having ABTS radical scavenging activity with IC_50_ values of 13.2 ± 0.03 *μ*g/mL for leaves and 120.2 ± 0.4 *μ*g/mL for fruit pulp in methanol extract [15]. Though previous studies have used methanolic extracts, in this study, ethanol/water was used to prepare the extract as the main focus was on the formulation of a supplement. In this regard, the Hex fraction of the fruit extract exhibited higher ABTS radical scavenging activity (EC_50_: 15.01 ± 0.65 *μ*g/mL) compared to the results reported in a previous study [15].

The differences observed among these studies are generally accepted, as the samples were from different geographic locations with different degrees of biotic and abiotic stress conditions that are associated with biosynthesis and expression of secondary metabolites [32].

In the FRAP assay, the reduction of the ferric-TPTZ complex to ferrous-TPTZ by accepting electrons from the sample was measured as the total amount of antioxidants. Compounds that exhibit the ferric reducing activity indicate that their ability to act as electron donors reduces the oxidized intermediates of the lipid peroxidation process. In this study, EA fraction of leaves reported the highest value among the tested fractions in leaves and fruits, which reveals the best-reducing property.

The ORAC assay is based on hydrogen atom transfer mechanism (HAT) and the inhibition of oxidation of fluorescein by peroxide radicals generated from 2,2'-azobis (2-amidino-propane) dihydrochloride (AAPH) with an antioxidant action. According to the present study, ORAC assay results implied that there is no correlation with other antioxidant assays. This fact still requires more supporting pieces of evidence as there is no available reported data on ORAC assay for the *S. caryophyllatum* fruit and leaf extracts.

The Fe^2+^ chelation assay is considered as an indirect antioxidant activity measurement which indicates the potential to inhibit Fenton reaction. The Fe^2+^ chelation was estimated quantitatively with ferrozine. The test procedure results in the formation of a red-colored complex with Fe^2+^ that reduces its color in the presence of a chelating agent. This is due to the interruption caused by the formed complex. Extracts and fractions exhibited the metal iron-chelating power, where the Hex fraction of fruits exhibited the highest iron chelation capacity. This is suggestive of the presence of bioactive molecules with chelating abilities in addition to the polyphenolics.

Strong antioxidant and antimicrobial potentials of the leaf oil of *S. caryophyllatum* along with GC-MS analysis have been reported for Indian plants by Soni et al. [33] and Nadarajan et al. [34]. The major compounds revealed by the present study are phytol, caryophyllene, humulene, globulol, and *α*-cadinol. The composition of leaf volatiles of *S. caryophyllatum* reported in this study relates and corresponds to those reported in the previous study. The previous study has reported 55 compounds in the winter season and 129 compounds in the summer season with promising antibacterial activity [34]. The major identified compound in winter essential oil was *α*-cadinol, and in summer, the essential oil was caryophyllene oxide where the phytol was the major compound in the present study [34]. Further, few common compounds such as *α*-cadinol, caryophyllene oxide, globulol, and phytol were also found.

Mineral deficiencies impose a major burden on public health and maintenance of healthy physiology. Therefore, supplementation is the best choice in order to fulfil the daily mineral requirement especially when an individual is suffering from a health complication such as DM. Some dietary minerals including Cr, Mg, Ca, Mn, K, Zn, and Se play a key role in physiological processes related to DM [35]. Magnesium deficiency is related to the risk of atherosclerosis, hypertension, cardiac arrhythmias, stroke, alterations in lipid metabolism, insulin resistance, metabolic syndrome, type 2 diabetes mellitus, osteoporosis, and depression.

Zinc is an important microelement with definite roles in metabolism and growth and it is also essential and vital for the smooth functioning of over 200 enzymes [36]. Zinc deficiency adversely affects the growth of T and B cells and apoptosis, growth retardation, and cognitive impairment [37]. Further, Zn is involved in the synthesis and secretion of insulin, and it is a structural part of Zn-dependent antioxidant enzymes such as superoxide dismutase [38]. Copper is an essential microelement that acts as a cofactor of many redox enzymes and as catalysts for iron absorption [36].

Chromium is an essential microelement for the biosynthesis of DNA, which regulates cell division and growth [39]. The Cr is also necessary for the regulation of normal glucose metabolism and the deficiency of Cr is related to impaired glucose tolerance [35]. Selenium plays a critical role in antioxidant defense due to its presence in the active center of antioxidative enzymes (glutathione peroxidase (GPX) and thioredoxin reductase (TrxRs)). This essential microelement impairs lipid peroxidation and protects cells against damage to genetic material [40]. A previous study carried out by Subramanian et al. [41] for the leaves of *S. caryophyllatum* reported having 99.275 ± 0.022 mg/kg, 17.302 ± 0.010 mg/kg, 45.498 ± 0.072 mg/kg, 47.344 ± 0.002 mg/kg, 7.634 ± 0.050 mg/kg, and 0.253 ± 0.003 mg/kg of Fe, Zn, Cu, Mn, Pb, and Cd, respectively [41]. The content of Na and K reported was absent and also Zn, significantly high Cu and low Mn levels and Cd were detected, and a higher amount of Pb was reported compared to the present study [41].

The results of the present study exhibited that the content of the mineral elements differs significantly among the parts of the similar plants. Furthermore, with comparison to the reported data, it reveals that the levels of mineral elements vary from different geographical locations due to the variation in soil types, agricultural and industrial activities, local growing conditions, plant interactions, and weather [8]. Nonetheless, studies have revealed that the metal-tolerant plants (metallophytes) possess the effects for phytoremediation through exposure to heavy metal stress showing varying degrees of therapeutically active constituents [42].

Thus, the risk analysis is required in plants before any preparation as the wild fruity plants are favored in controlling the growth and processing of the environment.

## 5. Conclusions

The present study concludes that the leaves and the fruits of *Syzygium caryophyllatum* plant possess antioxidant, antiamylase, and antiglycation activities and are a rich pool of essential mineral elements such as Zn, Mn, Cu, and Sr, which together may act to reduce the risk of associated diabetic complications and other noncommunicable diseases. Studies on isolation and characterization of the bioactive compound in leaves and fruit extracts are important for confirmation of the pharmacological properties of the responsible active compounds. Further, it can be concluded that *Syzygium caryophyllatum* leaves and fruits are potent natural sources that possess the potential to be developed into a natural supplement to aid the management process of type 2 diabetes mellitus.

## Figures and Tables

**Table 1:** The yield of *Syzygium caryophyllatum* plant extracts.

Scientific name	Part of plant	Extract	Weight of concentrated extract (g)	Yield %
*Syzygium caryophyllatum*	Leaves	CR	17.500	7.0
		Hex	<0.001	<0.1
		EA	0.114	0.1
		AQ	15.984	7.1
	Fruits	CR	31.200	62.0
		Hex	1.950	2.6
		EA	1.531	2.0
		AQ	10.165	13.6

CR = crude extract, Hex = hexane fraction, EA = ethyl acetate fraction, AQ = aqueous fraction.

**Table 2:** Total polyphenol content (TPC) and total flavonoid content (TFC) in different extracts of *Syzygium caryophyllatum*.

Scientific name	Part of plant	Extract	TPC (mg GAE/g of sample)	TFC (mg QE/g of sample)
*Syzygium caryophyllatum*	Leaves	CR	8.12 ± 0.46^a^	0.16 ± 0.01^a^
		EA	0.09 ± 0.01^b^	<0.01
		AQ	7.12 ± 0.16^c^	0.18 ± 0.02^ab^
	Fruits	CR	8.83 ± 0.62^ad^	2.02 ± 0.19^c^
		Hex	3.42 ± 0.29^e^	0.80 ± 0.05^d^
		EA	1.73 ± 0.76^f^	0.37 ± 0.01^e^
		AQ	2.16 ± 0.12^g^	0.10 ± 0.01^af^

Results expressed as mean ± standard deviation, CR = crude extract, Hex = hexane fraction, EA = ethyl acetate fraction, AQ = aqueous fraction. Mean values within a column superscripted by different letters are significantly different at *p* < 0.05.

**Table 3:** Antioxidant potentials of leaves and fruits of *Syzygium caryophyllatum*.

Scientific name	Part of plant	Extract	ORAC mg TE/g of extract	FRAP mg TE/g of extract	EC_50_ DPPH *μ*g/mL	EC_50_ ABTS *μ*g/mL	Iron chelation mg EDTA/g of extract
*Syzygium caryophyllatum*	Leaves	CR	221.00 ± 34.96^a^	48.27 ± 8.11^a^	500.20 ± 44.33^a^	107.93 ± 15.07^a^	33.2 ± 1.68^a^
		EA	344.31 ± 3.37^b^	262.26 ± 43.93^b^	91.20 ± 4.28^b^	40.61 ± 2.76^b^	36.01 ± 6.98^a^
		AQ	320.97 ± 20.62^bc^	73.42 ± 12.58^c^	251.05 ± 17.52^c^	27.04 ± 2.06^c^	56.17 ± 7.98^b^
	Fruit	CR	45.98 ± 2.58^d^	6.81 ± 1.17^d^	873.72 ± 12.52^d^	128.08 ± 6.12^d^	13.73 ± 0.69^c^
		Hex	32.77 ± 1.41^e^	53.91 ± 12.30^a^	100.97 ± 8.19^e^	15.01 ± 0.65^e^	74.82 ± 2.66^d^
		EA	118.65 ± 7.60^f^	63.07 ± 10.17^e^	135.53 ± 9.83^f^	89.54 ± 3.88^f^	8.28 ± 0.40^e^
		AQ	20.93 ± 4.49^g^	15.45 ± 0.68^f^	946.42 ± 65.04^g^	31.45 ± 1.45^g^	—

Results are expressed as mean ± standard deviation, *n* = 3. Mean values within a column superscripted by different letters are significantly different at *p* < 0.05. CR = crude, Hex = hexane, EA = ethyl acetate, AQ = aqueous. ORAC: oxygen radical absorbance capacity; FRAP: ferric reducing antioxidant power; ABTS: ABTS radical scavenging activity; DPPH: DPPH radical scavenging activity.

**Table 4:** The *α*-amylase inhibitory activity of isolated compound mixtures of Hex fraction of *Syzygium caryophyllatum* fruits (hexane and ethyl acetate solvents on isolation procedure).

Assay concentration *μ*g/mL	Inhibition %
200	57.63 ± 0.18^a^
100	55.99 ± 1.60^a^
50	57.30 ± 0.18^a^
45	23.39 ± 0.21^b^
40	22.45 ± 0.79^b^
35	21.43 ± 0.19^b^
30	14.33 ± 0.83^c^
25	12.78 ± 0.45^c^

Results are expressed as mean ± standard deviation, *n* = 3^∗^. Mean values within a column superscripted by different letters are significantly different at *p* < 0.05.

**Table 5:** The *α*-amylase inhibitory activity of hexane fraction and its chromatographic fraction of *Syzygium caryophyllatum* fruits.

Sample	IC_50_ (*μ*g/mL)
Hex fraction	47.20^a^ ± 0.30
Hex : EA, (0 : 100)	NLR
C_3_, DCM : EA (95 : 5)	2.27^b^ ± 1.81
Standard drug acarbose	87.96^c^ ± 1.43

Results are expressed as mean ± standard deviation, *n* = 3^*∗*^. NLR = no linear response. Mean values within a column superscripted by different letters are significantly different at *p* < 0.05. Hex = hexane, EA = ethyl acetate, DCM = dichloromethane, C_3_ = combined fraction 3.

**Table 6:** Antiglycation activity of crude extracts of *Syzygium caryophyllatum*.

Crude extract	% Inhibition			IC_50_ (*μ*g/mL)
	Concentration (*μ*g/mL)			
	50	100	200	
Leaves	54.88 ± 3.10^a^	79.87 ± 5.94^a^	93.61 ± 8.50^a^	57.83 ± 7.91
Fruits	41.31 ± 7.89^b^	56.66 ± 8.36^b^	47.97 ± 2.63^b^	—
Standard rutin		IC_50_ = 34.23 ± 3.18 *μ*g/mL		

Results are expressed as mean ± standard deviation, *n* = 3^∗^. Mean values within a column superscripted by different letters are significantly different at *p* < 0.05.

**Table 7:** Major compounds detected in *Syzygium caryophyllatum* leaf essential oil.

Peak	Compound	Formula and ^*∗*^mol wt	RT	Area %	SI	RSI
1	Caryophyllene	C_15_H_24_, 204,	14.79	3.21	954	954
2	Humulene	C_15_H_24_, 204,	16.45	3.36	907	907
3	*α*-Cadinol	C_15_H_26_O, 222,	27.81	3.87	923	928
4	Cyclosiolongifolene, 9,10-dehydro	C_17_H_26_O_2_, 202	28.26	3.82	790	800
5	Phytol	C_20_H_40_O, 296,	34.44	24.66	927	927

^*∗*^Mol wt = molecular weight.

**Table 8:** Mineral compositions of leaves and fruits of *Syzygium caryophyllatum*.

		Mineral elements (mg/kg per sample)														
		K	Fe	Na	Ca	Mg	Mn	Cu	Zn	Cr	Zn	Sr	Se	Pb	As	Cd
*Syzygium caryophyllatum*	Leaves	3559.25	221.98	4357.68	1843.52	2580.39	298.82	4.91	21.60	4.03	21.59	32.89	0.85	1.02	ND	ND
	Fruits	2944.28	49.55	756.21	149.27	855.55	18.14	5.23	7.75	4.65	7.75	2.70	0.09	0.15	ND	ND

RDA		3.5 g	9–15 mg	1.5 g	1 g	280–350 mg	50–60 *μ*g									

^*∗*^ND: not detected; limit of detection: 0.05 mg/kg; RDA: recommended daily intake.

## Data Availability

The data used to support the findings of this study are available from the corresponding author upon request.
